# Book Reviews

**Published:** 1917-09

**Authors:** 


### BOOK REVIEWS

---

**Books mentioned may be inspected at and ordered through this office. So far as possible, books received in any month will be reviewed in the issue of the second month following. Pamphlets, quarterly and similar periodicals, reports, transactions, etc., will, as a rule, merely be mentioned.**

---

**1916 Collected Papers of the Mayo Clinic,** Rochester, Minn. Octavo of 1014 pages, 411 illustrations. Philadelphia and London: W. B. Saunders Company, 1917. Cloth $6.50 net; Half Morocco $8.50 net.

This contains a series of articles on the stomach—gastric and duodenal ulcer, ordinarily so called, toxic gastric haemorrhage, duodenal ulcer with achlorhydria (free HCl absent) spasm of stomach and duodenum (X-ray studies), influence of gastric juice on jejunal mucosa, gastric ulcers following removal of adrenals, syphilis, tuberculosis, cancer, legitimate error in X-ray studies, etc. Several other articles relate to the biliary passages and the intestine. The uro-genital organs, ductless glands, blood, non-visceral surgery, pathologic and surgical technic and a section that is described as general, complete the work which is well indexed and provided with elaborate bibliographies. A book of such large size, covering many different subjects and mainly consisting of monographs of the utmost value, is obviously difficult to review satisfactorily. Its interest is by no means confined to surgeons.

---

**Practical Treatment,** Volume IV. By 76 eminent specialists. Edited by John H. Musser, Jr., M. D., Associate in Medicine, University of Pennsylvania; and Thomas C. Kelly, M. D., Instructor in University of Pennsylvania. Desk Index to the complete set of four volumes sent with this volume. Octavo 1000 pages, illustrated. Philadelphia and London: W. B. Saunders Company, 1917. Cloth, $7.00 net; Half Morocco, $8.50 net.

The present volume and index complete the work which was begun several years ago. Vol. 4 includes a general section, in which are articles on governmental prophylaxis, general principles of drug treatment, vaccines and sera, hydrology, Climatology, electrotherapy, Roentgen therapy, food and drug poisoning and poisoning by reptiles and insects, etc. The general sections following are devoted to infectious diseases, parasites, circulatory, respiratory, digestive, urinary and nervous systems (no distinction between system and apparatus), and constitutional diseases, a term that we are still unable to discontinue. It will be noted that this work is far beyond the ordinary alphabetic arrangement of brief and inadequate therapeutic measures and that it is more in line with reviews of periodic literature but with less strict time limits and freer scope to the authors of the various sections. While properly designated as a work on treatment, and of practical nature, neither term has been interpreted in a narrow sense. It is really a series of monographs, each as up-to-date and complete as possible and the only criticism that can be made is that, in a work of this volume and high aim, it is impossible to crystalize a progressive art and science or even to bring out at once, the entire assemblage of data representing the point reached by medical science at any given date. These impossibilities are inherent and inevitable and in no way detract from the usefulness of the work.

---

**The Elements of the Science of Nutrition.** By Graham Lusk, Ph. D., Sc. D., F. R. S., (Edin.), Professor of Physiology at Cornell Medical School, New York. Third Edition, Reset. Octavo of 641 pages, illustrated. Philadelphia and London: W. B. Saunders Company, 1917. Cloth $4.50 net.

This work deals with the physiologic and chemic principles on which dietetics rests and should be read as a foundation for the clinical direction of feeding, just as physiology, chemistry and pathology should be studied as a basis for intelligent therapeutics in general. The scope of the work can be judged from the titles of some of the chapters: Respiration Calorimeter, Starvation, Regulation of Temperature, N Equilibrium, Intermediary Metabolism and Respiratory Metabolism (Proteids), Influence of Ingestion of Fat, Intermediary and Respiratory Metabolism of Carbohydrates, Influence of Mechanical Work on Metabolism. The metabolism in various diseases is discussed in several chapters and a final chapter on food economics, though obviously not directly applicable under present abnormal conditions, is of great value.

---

**Personal Reminiscences of the N. Y. Hospital, 1856-1900,** Dr. Robert F. Weir, Attending Surgeon, 1876-1900, consulting surgeon thereafter, contained in the General Bulletin, published by the Society of the N. Y. Hospital, Vol. 1, No. 10.

This is a valuable and interesting contribution to medical history.

---

**Physical Exercises for Invalids and Convalescents.** Edward H. Ochsner, B. S., M. D., Chicago, C. V. Mosby Co., St. Louis. 54 pages, 40 illustrations, $0.75.

This is a series of mild exercises, not involving physical strain, and without apparatus of any kind. Each exercise is illustrated, an appropriate number of repetitions of the muscular movements is stated and directions as to control of posture are given. A work of this kind, far removed from athletics, aiming to correct defective carriage and to build up strength without over emphasizing muscular development, is adapted to many practical problems and should be generally used. It can be safely given to patients for instruction in details, the physician directing the general plan and degree of exercise and correcting faulty posture and failures to get the proper muscular swing.

---

**Practical Materia Medica and Prescription Writing.** Oscar W. Bethea, M. D., Ph. G., F. C. S., New Orleans, F. A. Davis Co., Philadelphia, 2d revised edition, 562 pages, $4.50.

Rather more than half of the work is devoted to materia medica, following the alphabetic arrangement, a moderate number of unofficinal remedies being included. Prescription writing is very thoroughly discussed, the tabulation of Latin terms and inflexions being especially convenient. Altogether too little attention is paid to metric prescribing. Incompatibility and solubility in water and alcohol and the medicolegal status of the prescription are well treated. The inclusion of fac similes of written prescriptions is an excellent point although it might be well to illustrate a greater variety of blanks and the use of the metric as well as the apothecary's system. An appendix devoted to discussions of prescription requirements, on the conference plan is especially valuable. An index of general indications for diseases gives a general idea of therapeutic applications.

---

**Hand Book of Anatomy,** James K. Young, M. D., F. A. C. S., Philadelphia, F. A. Davis Co., Philadelphia, 424 pages, 154 engravings, including colored plates. 5th revised edition, $2.

The free use of tables, the great clearness of the illustrations and the skillful avoidance of unnecessary details and prolix descriptions are the answer to the question as to how a comparatively small book could give so comprehensive and thorough an idea of anatomy. We recommend this book especially to men entering military service, for which portability is a prime requisite.

---

**Manual of the Diseases of the Eye,** Charles H. May, M. D., N. Y., Wm. Wood & Co., N. Y., 442 pages, 377 original illustrations, including 22 plates and 71 colored figures. 9th edition, revised, $2.50.

No better evidence of the value of this work can be given than the number of the edition and the fact that it has been translated into 7 foreign languages, including Japanese and Chinese. It is a thorough and accurate manual, not only abundantly and clearly but sensibly illustrated. That is to say, the illustrations follow the text and aid in comprehending statements and instructions in words and are not merely embellishments, more or less appropriate, as in some books.

---

**The Medical Clinics of North America.** Volume 1, Number 1, (The Johns Hopkins Hospital Number, July, 1917). Octavo of 193 pages, 14 illustrations. Philadelphia and London: W. B. Saunders Company, 1917. Published Bi-monthly. Price per year: Paper, $10.00; Cloth, $14.00.

We depart from our precedent in reviewing collective works, in specially mentioning Janeway's clinic on diabetes with associated failure of the external secretions of the pancreas, not only because of the intrinsic interest of the case but because it is a conference clinic, with inclusion of the report of a student, Mr. Rice, and the discussion as would naturally occur between attendant and consultant in the actual diagnosis and treatment of a difficult case.

